# Taxonomically distinct diatom viruses differentially impact microbial processing of organic matter

**DOI:** 10.1126/sciadv.adq5439

**Published:** 2025-05-02

**Authors:** Chana F. Kranzler, Devin A. Busono, Gweneth J. Walsh, Alyssa C. Carrillo, Kay D. Bidle, Kimberlee Thamatrakoln

**Affiliations:** ^1^ The Mina and Everard Goodman Faculty of Life Sciences, Bar-Ilan University, Ramat Gan, Israel.; ^2^ Department of Marine and Coastal Sciences, Rutgers University, New Brunswick, NJ, USA.

## Abstract

Phytoplankton viruses facilitate the production of dissolved organic matter (DOM) through host lysis, shaping DOM composition, and subsequent regenerative processing. We explored how DOM generated from a bloom-forming, centric diatom, infected with taxonomically distinct viruses—a single-stranded (ss) DNA and a ssRNA virus—impacted microbial processing of organic matter. DOM derived from uninfected and ssDNA virus–infected cultures supported growth in bacterial isolates and a mixed assemblage. In contrast, DOM from ssRNA virus infection did not stimulate growth, but rather induced ectoproteolytic activity, suggesting this DOM was less bioavailable. Exoprotease activity was also substantially higher in ssRNA virus–infected cellular exudates compared to ssDNA virus–infected and uninfected cultures. This suggests that DOM produced through virus-mediated host lysis does not a priori support secondary production and implicate ssRNA virus infection as a source of proteolytic activity in the water column, highlighting a multifaceted role for viruses in altering microbial utilization and remineralization length scales of organic matter in the ocean.

## Teaser

RNA virus infection alters the landscape of diatom organic matter, temporally decoupling the viral shunt and the microbial loop.

## INTRODUCTION

Phytoplankton contribute roughly half of the primary production on the planet (*1*), generating organic carbon that forms the base of marine food webs and fuels the biological carbon pump. Phytoplankton are also a primary source of dissolved organic matter (DOM) (*2*, *3*), shaping one of the largest reservoirs of carbon on the planet (*4*) via exudation, mortality, and cell lysis. Approximately 50% of phytoplankton-derived organic carbon comes into direct contact with heterotrophic bacterial communities (*5*), fueling secondary production and rigorous elemental cycling within the microbial loop (*6*). As obligate osmotrophs, heterotrophic bacteria rely on the cellular transport of low molecular weight DOM (<600 Da) from the surrounding environment (*7*). Because these compounds are often not readily available at sufficient concentrations, bacteria use cell surface–bound (ecto-) hydrolytic enzymes that catalyze the processing and breakdown of complex, high molecular weight DOM into smaller, transport-ready polymers (*8*–*10*). Ectohydrolytic enzyme activity varies over spatial and temporal scales in the ocean (*8*, *11*, *12*) and is generally considered a rate-limiting step for DOM utilization by bacteria (*13*).

Lytic viral infection is considered a “catalyst” of DOM production in the ocean (*14*). With ~10^7^ viruses per milliliter of seawater (*15*), marine viruses are estimated to turnover a quarter of photosynthetically fixed carbon daily, supplying microbial communities with DOM released during host lysis (*16*). This process, known as the “viral shunt,” redirects the biogeochemical flux of carbon and associated elements toward microbial assimilation and remineralization and away from trophic transfer or export via the biological carbon pump (*16*). In marine phytoplankton, viral infection can be influenced by a range of environmental factors such as nutrient availability (*17*–*19*), temperature (*20*, *21*), and light (*22*, *23*). Virus infection also results in substantial reprogramming of host metabolism (*24*), toward an infected cell state, or virocell (*25*), generating a unique, metabolic signature released during host lysis (*26*–*28*). This suggests that in addition to increasing DOM supply, virus-induced alterations in DOM composition could differentially influence downstream biogeochemical cycling.

In this study, we characterized microbial responses to DOM from a marine diatom infected by taxonomically distinct viruses. Diatoms are among the most widespread phytoplankton in the ocean and are key players in the carbon cycle, contributing ~20% of global primary production (*29*). As obligate silicifiers, the ballasted silica-based cell wall effectively transports diatoms to depth, substantially contributing to carbon export (*30*). However, microbial remineralization of diatom silica (*31*) in the upper ocean weakens the transfer efficiency and export of diatom organic carbon and associated elements into the mesopelagic (*32*). The relative contribution of dissolved silicon to new diatom production [dissolution-to-production (D:P) ratio] can be as high as 70% in some regions of the ocean (*32*), highlighting the importance of regenerative silicon in sustaining diatom communities. Observations of high-biomass, low-export systems (*33*), together with high spatial and temporal variability in the remineralization and recycling of diatomaceous material (*32*, *34*), underscore the importance of resolving the factors that determine the fate of diatom organic matter in the water column. While far less studied than other phytoplankton-infecting viruses (e.g. cyanophage, *Phycodnaviridae*), it is becoming increasingly clear that diatom viruses are active players in community dynamics (*35*). Using a model single-stranded RNA (ssRNA) virus (family *Marnaviridae*) and single-stranded DNA (ssDNA) virus (family *Bacilladnaviridae*) infecting a single diatom host, together with two diatom-associated marine bacterial isolates and a natural bacterial assemblage, we explored how DOM produced by infected diatoms affects subsequent microbial utilization and regenerative processing.

## RESULTS

**Fig. 1.**
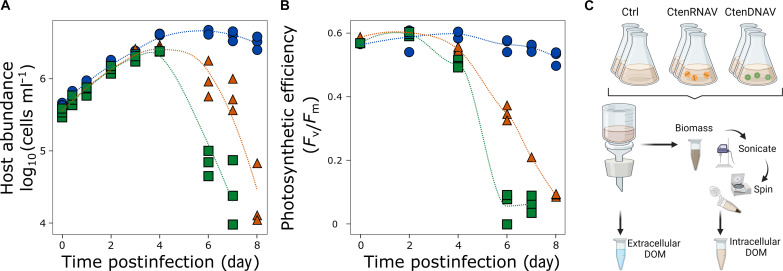
Host-virus infection dynamics in *C. tenuissimus* and sample collection. (**A**) Host abundance (in cells per milliliter) and (**B**) photosynthetic efficiency (*F*_*v*_/*F*_*m*_) in uninfected cultures (blue circles) and those infected with CtenRNAV (orange triangles) or CtenDNAV (green squares). Biological triplicates are plotted with a line depicting a local polynomial regression (LOESS). (**C**) Cultures were fractionated into extracellular DOM (eDOM, <0.22 μm) and biomass (>1.2 μm) samples by filtration. Biomass was further partitioned into particulate and intracellular DOM (iDOM) using freeze-thaw sonication cycles and centrifugation (see the Materials and Methods). Created in BioRender by C. Kranzler (2024; https://BioRender.com/yp73w5l).

Cultures of the bloom-forming centric diatom, *Chaetoceros tenuissimus* strain 2-10, were infected by one of two taxonomically distinct viruses—the ssRNA virus, *Chaetoceros tenuissimus* RNA virus (CtenRNAV) type I (*36*), and the ssDNA virus, *Chaetoceros tenuissimus* DNA virus (CtenDNAV) type I (*37*)—alongside uninfected control (Ctrl) cultures. A decrease in host concentration (Fig. 1A) and photosynthetic efficiency (*F*_v_/*F*_m_; Fig. 1B) was observed in infected cultures 4 days postinfection (dpi), with CtenDNAV-infected cultures exhibiting a more rapid decline in both metrics. Near complete lysis was observed 7 and 8 dpi for CtenDNAV- and CtenRNAV-infected cultures, respectively (Fig. 1, A and B). Throughout the course of infection, extracellular DOM (eDOM; <0.22-μm filtrates) and cellular biomass were collected for downstream analyses (Fig. 1C), with the latter used to generate intracellular DOM (iDOM) via mechanical lysis (see Materials and Methods).

### DOM generated during virus infection differentially regulates bacterial growth and ectoproteolytic activity

**Fig. 2.**
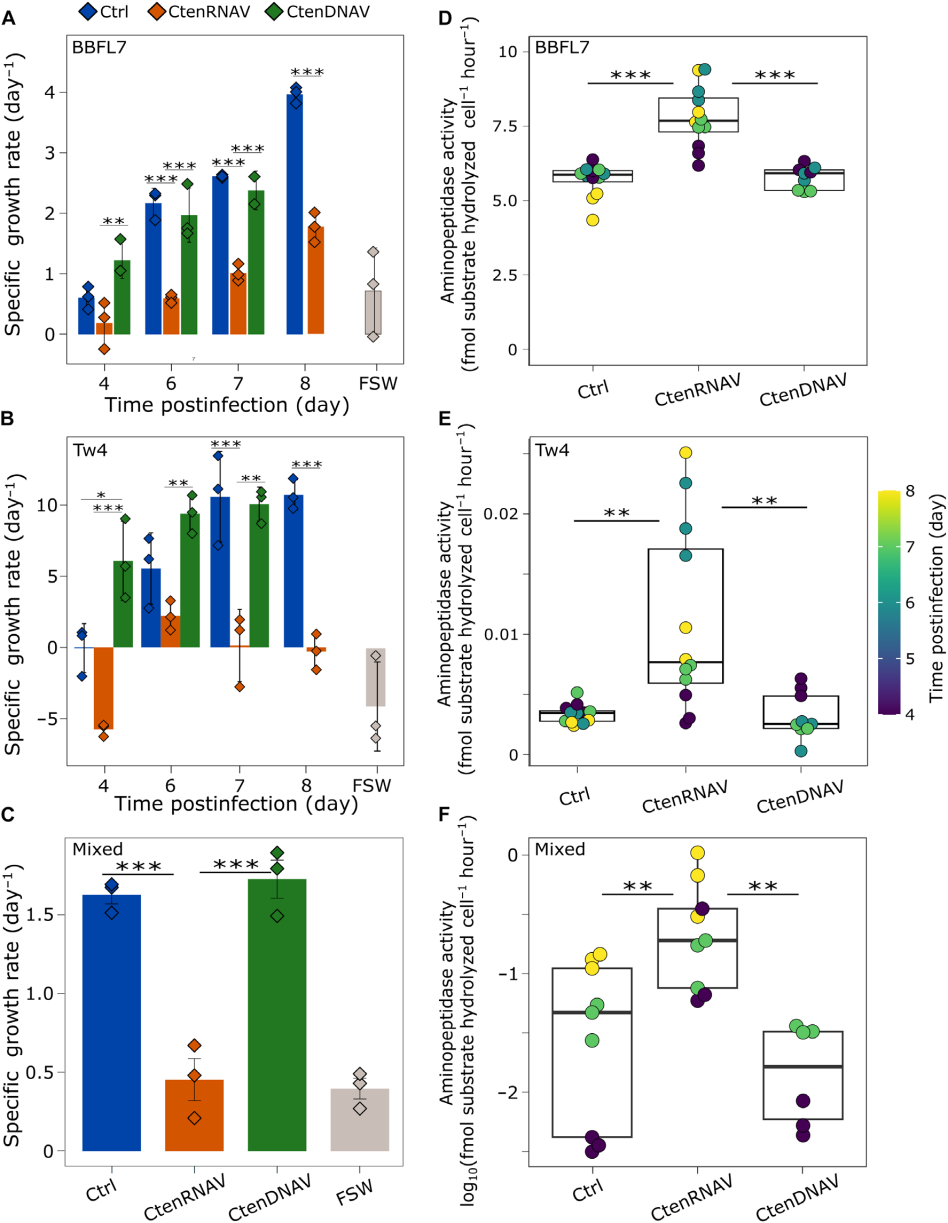
Bacterial response to diatom-derived eDOM generated during viral infection. Specific growth rates (μ; per day) of (**A**) BBFL7 (**B**) Tw4, and (**C**) a coastal bacterial assemblage (mixed) following incubation with eDOM collected from uninfected (Ctrl; blue), CtenRNAV-infected (orange), and CtenDNAV-infected (green) *C. tenuissimus* cultures or FSW (gray). Mean and SE of triplicate incubations are shown along with individual replicates (diamonds). Data are representative of two to three independent experiments. Cell-specific aminopeptidase activity (in femtomoles of substrate hydrolyzed per cell per hour) in (**D**) BBFL7, (**E**) Tw4, and (**F**) a coastal bacterial assemblage upon exposure to eDOM fractions from Ctrl, CtenRNAV-, and CtenDNAV-infected cultures. Color scale denotes time postinfection (in days) of eDOM sample collection throughout each experiment. Boxes depict median (line), upper, and lower quartiles; whiskers denote values 1.5× the interquartile range. Two-way analysis of variance (ANOVA) (A and B) and ANOVA (C to F) with Tukey’s post hoc test for pairwise comparison, ***P < 0.001, **P < 0.01, and *P < 0.05. See table S1 for a summary of statistical analyses.

The microbial response to diatom-derived DOM was tested with two previously isolated bacteria—a *Sphingobacterium-Flavobacterium* sp. (BBFL7) and a γ*-Proteobacterium, Alteromonas* sp. (Tw4) (*38*, *39*). Both genera are implicated in the colonization, utilization, and remineralization of diatom-derived organic matter (*39*–*41*). In incubations with diatom-derived eDOM, specific growth rates (μ; per day) of BBFL7 and Tw4 were positively correlated with time (fig. S1), increasing from day 4 to day 8 in all treatments (Ctrl, CtenDNAV, and CtenRNAV), indicating the presence of DOM that could support bacterial growth. eDOM derived from Ctrl and CtenDNAV-infected cultures stimulated faster growth of both BBFL7 and Tw4 compared to filtered seawater (FSW; *P* < 0.001), while eDOM from CtenRNAV-infected cultures did not (*P* > 0.5; Fig. 2, A and B). Similar findings were observed with a freshly collected, coastal, bacterial assemblage (Fig. 2C). The differential microbial growth response was not due to total dissolved organic carbon (DOC) load in eDOM samples, as there was no discernable difference in DOC concentration between treatments on day 4 or 6 of the infection experiment (*P* = 0.09; fig. S2A). Optical analysis of eDOM yielded a higher spectral slope ratio (*S*_*R*_) for CtenDNAV-derived eDOM (*S*_*R*_ = 0.127 ± 0.020) compared to both Ctrl (*S*_*R*_ = 0.071 ± 0.012) and CtenRNAV-derived (*S*_*R*_ = 0.071 ± 0.014) eDOM (fig. S2B), indicative of a greater fraction of low molecular weight compounds in CtenDNAV-derived eDOM (*42*). Together, these measurements suggest that the observed virus-specific impacts on bacterial growth are due to fundamental differences in DOM quality rather than quantity.

The inability of CtenRNAV-derived eDOM to stimulate bacterial growth (Fig. 2, A to C) suggested that this DOM pool was not readily bioavailable and may require additional processing before utilization. Proteins account for ~50% of organic carbon in marine microbes (*3*) and ectoproteolytic activity, in particular, can exceed other ectohydrolytic enzymes by two to three orders of magnitude (*11*, *12*), resulting in substantial degradation of organic matter in the water column (*10*). Both BBFL7 and Tw4 were previously shown to exhibit ectoproteolytic activity upon incubation with diatom-derived detritus, with BBFL7 displaying markedly high cell-specific ectoprotease activity (*39*). Using the fluorogenic substrate, leucine-7-amino-4-methylcoumarin (Leu-AMC), as a proxy for microbial processing of organic matter, we found that cell-specific aminopeptidase activity was significantly stimulated after exposure to CtenRNAV-derived eDOM, compared to Ctrl and CtenDNAV-derived eDOM, increasing ~1.4 and ~3-fold in BBFL7 and Tw4, respectively (Fig. 2, D and E). Enhanced aminopeptidase activity after exposure to CtenRNAV-derived eDOM was also observed in a freshly collected, coastal, bacterial assemblage (Fig. 2F).

**Fig. 3.**
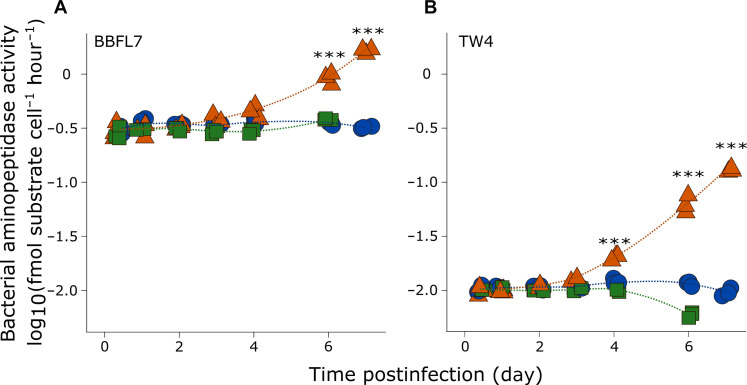
Bacterial ectoproteolytic activity in response to iDOM generated during virus infection. Cell-specific aminopeptidase activity (in femtomoles of substrate hydrolyzed per cell per hour) in (**A**) BBFL7 and (**B**) Tw4 upon exposure to iDOM collected from uninfected *C. tenuissimus* cultures (blue circles) and cultures infected with CtenRNAV (orange triangles) and CtenDNAV (green squares) throughout the time course of viral infection. Biological triplicates are plotted with a line depicting a local polynomial regression (LOESS).Two-way ANOVA with Tukey’s post hoc test for pairwise comparison, ***P < 0.001. Data are representative of two to three independent experiments. See table S1 for a summary of statistical analyses.

As eDOM is composed of cellular exudates and compounds that ultimately derive from iDOM, we tested whether iDOM from infected cells (Fig. 1C) could also differentially stimulate bacterial ectoproteolytic activity. Cell-specific aminopeptidase rates in both BBFL7 and Tw4 cultures were considerably enhanced following exposure to CtenRNAV-derived iDOM, compared to Ctrl- and CtenDNAV-derived iDOM (Fig. 3) with a ~2-fold stimulation in cellular aminopeptidase rates in BBFL7 by 6 dpi, which increased to ~5-fold by 7 dpi (Fig. 3A). Although cell-specific aminopeptidase activity in Tw4 was substantially lower than BBFL7, as expected (*39*), a similar stimulatory response to CtenRNAV-derived iDOM was observed, with a ~2-fold stimulation in activity by 4 dpi that increased to ~8-fold by 6 dpi and ~11-fold by 7 dpi (Fig. 3B). Without added bacteria, background aminopeptidase rates in iDOM samples were ~10-fold lower, confirming that the observed response to CtenRNAV-derived iDOM was attributed to BBFL7 and Tw4 (fig. S3).

To further discern between virus-specific impacts on iDOM and a general diatom cell death response, we tested bacterial ectoproteolytic activity in response to iDOM collected from senescent *C. tenuissimus* cultures (fig. S4A). Exposure to this iDOM did not stimulate activity in BBFL7, yielding similar rates to those measured in response to iDOM collected from cultures during exponential and stationary growth phases (fig. S4B). In Tw4, aminopeptidase activity in response to iDOM from senescent cultures was fourfold higher than exponential- and stationary-phase cultures; however, these rates were ~40% lower than those measured in response to CtenRNAV-derived iDOM at 6 dpi (fig. S4C), demonstrating that a substantial portion of the induced ectoproteolytic activity was specific to CtenRNAV infection.

### RNA virus–infected diatoms are an additional source of free, active exoproteases

**Fig. 4.**
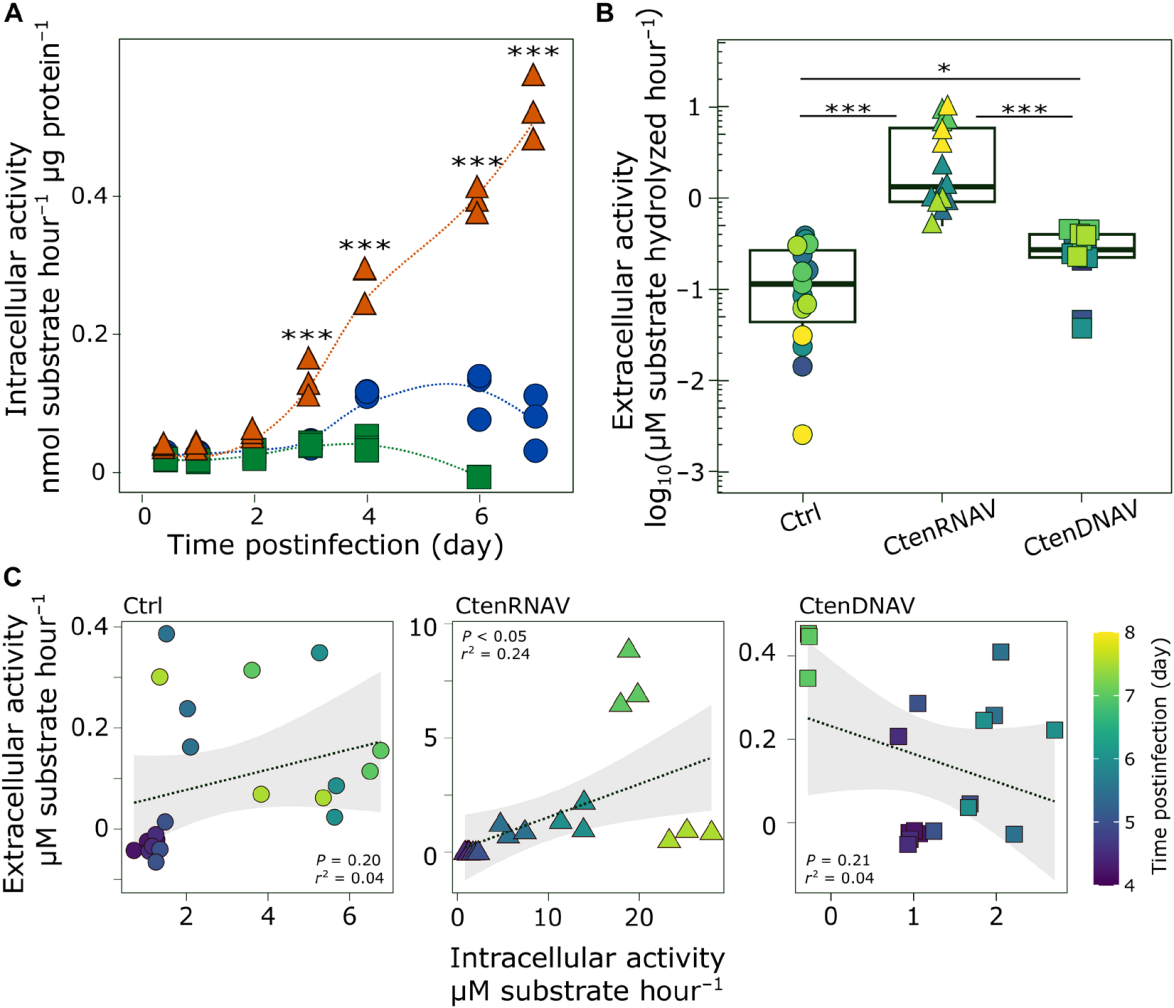
Protease activity in *C. tenuissimus* during virus infection. (**A**) Intracellular aminopeptidase activity (in nanomoles of substrate hydrolyzed per hour per microgram of protein) in *C. tenuissimus* iDOM samples collected throughout a time course of infection with CtenRNAV (orange triangles) and CtenDNAV (green squares) compared with uninfected (Ctrl) cultures (blue circles). Biological triplicates are plotted with a line depicting a local polynomial regression (LOESS).Two-way ANOVA, Tukey’s post hoc test, ***P < 0.001. (**B**) Free, dissolved, extracellular aminopeptidase activity (in micromolar of substrate hydrolyzed per hour) measured in cellular exudates (eDOM; <0.22 μm) collected from *C. tenuissimus* infected with CtenRNAV and CtenDNAV compared to uninfected cultures (Ctrl). Boxes depict median (line), upper, and lower quartiles; whiskers denote values 1.5× the interquartile range. ANOVA, Tukey’s post hoc test, ***P < 0.001 and *P < 0.05. (**C**) Relationship between intracellular and extracellular aminopeptidase activities (in micromolar of substrate hydrolyzed per hour) measured in Ctrl (left), CtenRNAV (middle), and CtenDNAV (right) treatments. Color scale in (B) and (C) denotes time of sample collection throughout each infection experiment. Line of the best fit with 95% confidence intervals (gray shading) shown describing linear regression analysis with *r*^2^ and *P* values depicted for each fit. See table S1 for summary of statistical analyses.

The onset of physiological stress and programmed cell death in phytoplankton have both been previously linked with enhanced intracellular protease activity (*43*, *44*). Consistent with these studies, there was a ~4- and ~9-fold increase in intracellular aminopeptidase activity in stationary and senescent *C. tenuissimus* compared to exponentially growing cultures (fig. S4D). CtenRNAV-infected cultures exhibited even higher intracellular aminopeptidase activity than senescent cells, increasing ~12-fold by 4 dpi and ~23-fold by 7 dpi (Fig. 4A). CtenDNAV infection did not result in a marked increase in intracellular aminopeptidase activity compared to Ctrl cultures (Fig. 4A). Active exo- (free, dissolved) proteases were also detected in Ctrl and infected exudates by 3 dpi (Fig. 4B). Activity in CtenRNAV exudates ranged from 4- to 38-fold higher than Ctrl and CtenDNAV exudates, with a maximal increase at 6 dpi (Fig. 4B). Moreover, in CtenRNAV-infected cultures, exoproteolytic activity was positively correlated with intracellular aminopeptidase activity (*P* < 0.05, *r*^2^ = 0.24; Fig. 4C), suggesting that the functional, dissolved exohydrolytic enzyme pool was derived from intracellular proteases produced during CtenRNAV infection. In contrast, no correlation was detected between extracellular and intracellular activities in Ctrl or CtenDNAV treatments (Fig. 4C and table S1).

## DISCUSSION

### Differentially regulated microbial processing of DOM by taxonomically distinct diatom viruses

**Fig. 5.**
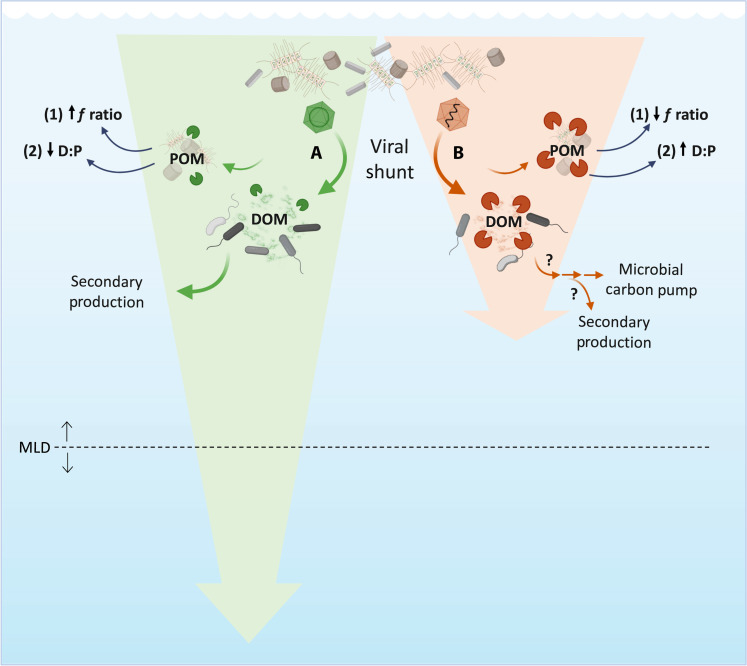
Conceptual model of diatom virus-specific impacts on microbial processing and remineralization length scales. Virus infection and virus-mediated host lysis in diatoms lead to the release of virus-specific DOM via the viral shunt. The DOM pool generated by ssDNA virus infection (left side, in green) directly supports microbial growth, fueling secondary production. In contrast, DOM derived from ssRNA virus infection (right side, in orange) is not readily available and temporally decouples the viral shunt from microbial production. Enhanced proteolytic activity (pac-man symbols) in response to ssRNA virus infection differentially affects the breakdown of particulate organic matter (POM), leading to an increase in (1) regenerated nitrogen, reducing the *f*-ratio, and (2) silica dissolution, increasing D:P, ultimately shortening the remineralization length scale (RLS) of particulate material as compared to ssDNA virus infection (downward background arrows). Proteolytic cleavage of DOM may eventually support secondary production or could end up in the recalcitrant pool fueling the microbial carbon pump. The lack of stimulation in enzymatic activity in response to ssDNA virus infection would limit the availability of regenerated nitrogen and silicon, increasing the *f*-ratio (1) and decreasing D:P (2), respectively. The relationship of the RLS to the mixed layer depth (MLD; dashed horizontal line) will ultimately determine the relative coupling of viruses to recycling (shunt) and export (shuttle). Created in BioRender by C. Kranzler (2024; https://BioRender.com/qlk4ies).

Our findings demonstrate that the microbial response to diatom-derived DOM is dramatically shaped by virus infection, such that taxonomically distinct viruses impose specific and differential impacts on downstream microbial processes (Fig. 5). The breakdown and utilization of DOM are affected by both quantity and composition, ultimately shaping the bacterial community response, structure, and function (*8*, *45*). Virus infection in phytoplankton has been shown to generate a unique DOM pool (*26*–*28*), and recent work on cyanophage demonstrated enhanced microbial growth and altered community structure in response to viral lysates (*46*, *47*). Here, in the bloom-forming, centric diatom *C. tenuissimus*, we show that DOM produced during RNA virus infection did not support bacterial growth but stimulated bacterial-mediated ectoproteolytic activity. In contrast, DOM produced during ssDNA virus infection supported bacterial growth but did not stimulate ectoproteolytic activity. Although it is possible that CtenRNAV-derived DOM may support secondary production in other types of bacteria, our experiments with phylogenetically and metabolically distinct bacteria (*38*, *39*), together with a coastal bacterial assemblage, demonstrate that viral infection of diatoms can differentially affect microbial utilization of DOM. Thus, in addition to differential impacts on host metabolism, different viruses infecting a single host can imprint unique signatures on diatom-derived DOM that influence its bioavailability and processing, altering the landscape of phytoplankton-derived organic matter available for heterotrophic bacterial growth and secondary production.

Although the exact trigger for the stimulation of bacterial ectoprotease activity by CtenRNAV-derived DOM is unknown, prior work shows that ectoenzyme activity can be regulated by a range of factors, including substrate concentration (*48*, *49*), growth phase or starvation (*50*), and metal cofactor availability (*51*). High ectoenzyme activity in mesopelagic waters has also been invoked as a mechanism enabling resident bacteria access to the more recalcitrant DOM present at these depths (*52*), supporting a relationship between DOM bioavailability and ectoenzyme activity. However, lability does not fully explain this relationship, as the presence of high–molecular weight compounds in DOM derived from uninfected cultures (inferred from spectral slope analysis) did not stimulate bacterial activity. One explanation could be the additional presence of specific elicitors of ectoenzyme activity, such as polyunsaturated aldehydes (*53*). In a recent study, we found that CtenRNAV infection generates a suite of dissolved oxylipins (a class of polyunsaturated aldehydes) that were not produced by uninfected or CtenDNAV-infected cultures (*54*). Several of these oxylipins are known to exert inhibitory effects on bacterial growth (*54*), which may further contribute to the reduced bacterial growth response to CtenRNAV-derived DOM. Together, our findings suggest that CtenRNAV infection results in the production of a unique DOM pool that stimulates a microbial, ectoproteolytic response that would facilitate the breakdown of DOM into transport-ready, utilizable material.

In addition to stimulating bacterial-mediated ectoproteolytic activity, CtenRNAV infection also resulted in increased intracellular aminopeptidase activity and the release of active exoproteases. Many viruses either directly encode for proteases and/or recruit host proteases, inducing intracellular modifications that facilitate viral replication, immune evasion, and host shutdown (*55*, *56*). While CtenRNAV infection may activate a variety of unique host proteases, the CtenRNAV genome also encodes a putative cysteine protease within the replicase polyprotein (*57*), similar to other viruses in the order *Picornavirales* (*58*), which use this protease in various aspects of the virus life cycle, including viral polyprotein processing, inhibition of host transcription, and the induction of apoptosis (*59*). No putative proteases have been identified within the CtenDNAV genome, suggesting that enhanced proteolytic activity may be specific to RNA virus infection.

These observations suggest that RNA virus infection specifically constitutes an additional source of free, active, dissolved exoproteases. Exohydrolytic enzymes are increasingly regarded as mechanistically important in the breakdown and structuring of organic matter in the water column (*8*, *60*). In marine systems, the ratio of exo- (free) to ecto- (cell-associated) enzymes can vary substantially (*61*) with exoenzyme production stemming from a range of processes, including microbial stress and mortality (*60*), substrate availability (*50*), or mechanical lysis by grazers (*62*). While heterotrophic bacteria are considered a primary source of free hydrolytic enzymes, exoenzymes can also originate from phytoplankton. In diatoms, *Thalassiosira weissflogii* exhibited increased intracellular protease production in response to both light and nitrogen limitations (*43*), while *Chaetoceros didymus* released proteases upon exposure to algicidal bacteria (*63*), highlighting that both physiological stress and pathogen defense are regulators of diatom exoprotease production. These data also provide additional context for prior observations documenting a correlation between RNA virus–mediated mortality and extracellular proteolytic activity during the spring diatom bloom in the California Current Ecosystem (*17*) and suggest that enhanced proteolysis may serve as a metabolic fingerprint of RNA virus infection.

The development and application of cultivation-independent approaches, including amplicon sequencing, viromics, and meta-genomics/transcriptomics (*17*, *64*–*66*), have demonstrated that RNA viruses are ubiquitously distributed throughout the global ocean and may rival double-stranded DNA viruses in abundance (*67*). Several studies also suggest that even the more elusive ssDNA viruses—subjected to technical bias by some sequencing approaches (*68*)—are widespread in marine systems (*69*) and, at times, can constitute a significant fraction of the marine virome (*70*). While both virus types are likely involved in determining the fate of diatom organic matter, the relative contribution of different viruses within a diverse diatom community is not yet known. The characterization here of a virus-specific relationship with downstream microbial growth and enzymatic activity further emphasizes the importance of future observational and experimental studies that better constrain the prevalence and biogeochemical impacts of both RNA and ssDNA virus infections in diatoms.

### Decoupling the viral shunt from microbial production

The viral shunt describes a classical paradigm in which virus-mediated host lysis redirects the flow of organic matter away from higher trophic levels and directly fuels the microbial loop and secondary production (*16*). Our laboratory-based findings demonstrate that DOM released by virus-mediated lysis is not a priori bioavailable and compatible for microbial consumption, providing a conceptual framework that challenges the assumption that the viral shunt is a direct conduit for microbial production. Rather, some viruses can temporally decouple the viral shunt from microbial growth, generating a lag in secondary production through the release of a distinct DOM pool that is not immediately utilizable to the prevailing bacterial consortium (Fig. 5) and potentially fueling the microbial carbon pump (*71*).

The role of viruses in mediating carbon export via the viral shuttle has been a focus of intense study in recent years (*72*–*74*). We posit that virus-mediated stimulation of bacterial ectoproteases, concomitant with the release of active exoproteases from infected diatoms, constitutes an additional role for viruses in mediating the hydrolytic transition of organic matter from particulate to dissolved, thereby altering the remineralization length scale (RLS; Fig. 5). Mechanistic control of microbial-mediated hydrolysis of phytoplankton-derived organic matter has been similarly implicated in structuring the RLS (*53*, *75*). Ultimately, the relationship of the RLS to the mixed layer depth will determine the relative coupling of viruses to recycling (shunt) and export (shuttle). In diatoms, virus infection can result in particle aggregation facilitated by the production of sticky, proteinaceous Coomassie-stainable particles (*76*), which, when ballasted by biogenic silica, would favor the viral shuttle. However, hydrolysis of the protective organic matrix surrounding the diatom frustule by bacterial ectoproteases leads to rapid silica dissolution (*31*). This loss of biogenic silica would substantially weaken the mineral ballast of sinking aggregates and reduce the efficacy of the viral shuttle, shortening the RLS and enhancing the supply of regenerative Si to resident diatoms in the surface ocean (Fig. 5). In parallel, preferential processing of nitrogen-rich, proteinaceous material within marine snow aggregates relative to carbon-rich macromolecules such as polysaccharides (*10*, *77*) would augment the supply of regenerated nitrogen and decrease the *f*-ratio (Fig. 5). Thus, virus-stimulated bacterial ectoproteolysis may help explain widespread observations of high Si D:P ratios (*32*) and elevated C:N ratios in sinking marine snow (*78*). Collectively, our findings mechanistically and biogeochemically link diatoms, viruses, and bacteria, highlighting the dynamic and multifaceted impacts of virus infection on the fate of organic matter in the ocean.

## MATERIALS AND METHODS

### Diatom culturing conditions and infection experiments

*C. tenuissimus* Meunier strain 2-10, isolated from the coastal waters of Japan, and its associated viruses were provided by Y. Tomaru at the National Research Institute of Fisheries and Environment of Inland Sea, Japan. Nonaxenic host cultures were maintained in modified SWM-3 medium with 0.2 mM silicate, 2 mM nitrate, and 2 nM Na_2_SeO_3_ at 15°C on a 12:12 light:dark cycle at ~120 μmol of photons m^−2^ s^−1^ (*17*). Cell abundance was measured on a BD Accuri C6 flow cytometer using chlorophyll fluorescence and forward scatter. Resident bacteria associated with the culture were quantified by SYBR staining and flow cytometry and found to range from 5 × 10^4^ to 3 × 10^5^ cells ml^−1^, two to three orders of magnitude lower than the bacterial concentrations used in growth experiments and enzyme assays. Virus stocks were generated by filtering infected cultures through a 0.22-μm pore size filter to remove cellular debris and resident bacteria and stored at 4°C. The abundance of infectious virus particles (in infectious units per milliliter) was measured using the most probable number assay (MPN) and calculated using the Environmental Protection Agency MPN calculator (*17*). Cultures were infected at mid-exponential phase (~2 × 10^5^ to 5 × 10^5^ cells ml^−1^) at a multiplicity of infection of 10.

### Biophysical measurements

Maximum photochemical quantum yield of photosystem II (photosynthetic efficiency; *F*_v_/*F*_m_) was measured using a custom-built fluorescence induction and relaxation system (*79*), providing measurements of *F*_o_ (minimum) and *F*_m_ (maximum) fluorescence yields. Maximum efficiency of photosystem II was calculated as *F*_v_/*F*_m_ = (*F*_m_ − *F*_o_)/*F*_m_.

### Sample collection and processing

DOM samples were collected daily during the time course of infection. Cellular exudates, or eDOM, were first filtered through a 1.2-μm polycarbonate filter (Millipore), followed by a 0.2-μm polyvinylidene difluoride filter to remove smaller particulates and resident bacteria and stored in aliquots at −80°C before use. Cell biomass was collected onto 1.2-μm polycarbonate membrane filters, resuspended in 0.2-μm FSW, and pelleted by centrifugation at 4000*g* for 10 min at 4°C. After removing the supernatant, pellets were flash frozen in liquid N_2_ and stored at −80°C until processing. To generate iDOM, pellets were thawed on ice, resuspended in 500 μl of FSW, flash frozen in liquid N_2_, thawed, and then sonicated for 30 s using a Misonex probe sonicator set to a power setting of 2. This freeze-thaw, sonication cycle was repeated two more times before centrifuging at 10,000*g* at 4°C for 15 min. Supernatants, representing the iDOM fraction, were transferred to fresh tubes and stored at −80°C.

### Protein quantification, total organic carbon analysis, and absorbance spectra

Total protein concentration was measured for each iDOM sample using a Pierce 600-nm protein assay (Thermo Fisher Scientific), alongside a bovine serum albumin standard curve (Pierce) in a SpectraMax M3 microplate reader (Molecular Devices). Final protein concentrations in iDOM extracts were linearly correlated with the total number of cells collected in each sample (*r*^2^ = 0.82, *P* < 0.001, *r* = 0.91; fig. S5), providing a quantitative measure of mechanical lysis and indicating a similar extraction efficiency across all samples. Total organic carbon (TOC) in eDOM samples was measured at the New Jersey Institute of Technology Material Characterization Lab (Newark, NJ). The spectral slope ratio (*S*_*R*_) of eDOM samples were measured in a 1-cm quartz cuvette on a SpectraMax spectrofluorometer (Molecular Devices) and calculated according to Helms *et al.* (*42*). Briefly, a linear regression of the log-transformed absorbance spectra was used to calculate the slope between 275 and 295 nm (*S*_275–295_) and 350 and 400 nm (*S*_350–400_). *S*_*R*_ represents the ratio of *S*_275–295_ to *S*_350–400._

### Bacteria culturing conditions and growth

Bacterial isolates BBFL7 and Tw4—previously isolated from a diatom bloom or following incubation with diatom detritus (*38*, *39*)—were grown from glycerol stocks and maintained on Zobell 2216E agar plates at 15°C (*39*). Before each experiment, a single colony was inoculated in liquid Zobell medium and grown for several days at 15°C with shaking (~100 rpm). Growth was monitored using optical density at 600 nm (SpectraMax M3 microplate reader) and calibrated to cell abundance measurements by flow cytometry. For flow cytometry–based analysis, bacteria were fixed in 0.5% glutaraldehyde with an incubation for 20 min at 4°C, followed by flash freezing in liquid N_2_ and storage at −80°C before analysis. Samples were stained with a 1:20,000 dilution of SYBR Gold stock (Molecular Probes) in Tris-EDTA buffer for 10 min and then analyzed on a BD Accuri C6 Plus flow cytometer using side scatter and fluorescence (488-nm excitation and 525-nm emission). For bacterial growth incubation experiments with diatom-derived eDOM, bacterial cultures were harvested in logarithmic growth phase, spun down at 10,000*g* for 2 min at 15°C, and resuspended in either FSW or eDOM samples at a starting cell density of ~10^7^ and ~10^8^ cells ml^−1^ for BBFL7 and Tw4, respectively. For experiments with natural bacterial communities, whole seawater was collected from the Rutgers University Marine Field Station located on the Mullica River Great Bay estuary, filtered through a borosilicate glass fiber filter (GF/F), and stored at 4°C until use (less than 1 week after collection). Specific growth rates (μ; per day) were calculated during logarithmic growth according to the following equation, which incorporates the natural log of bacterial cell concentration (*C*) over a specific interval of time (*t*):

$$\mu =\frac{\text{ln}C_{2}-\text{ln}C_{1}}{t_{2}-t_{1}}$$

### Measurements of protease activity

Exponentially growing cultures of BBFL7 or Tw4 were harvested by centrifugation at 10,000*g* for 2 min at 15°C. The supernatant was discarded, and bacteria were resuspended in FSW. For iDOM incubation experiments, a final concentration of protein (5 μg ml^−1^) was incubated with bacteria at a cell concentration of ~10^7^ cell ml^−1^ (BBFL7) and ~10^8^ cell ml^−1^ (Tw4). For eDOM experiments, bacteria were added directly to eDOM at the same concentrations used for iDOM experiments. Aminopeptidase activity was measured using the fluorogenic substrate Leu-AMC by transferring 200 μl of each sample in triplicate (DOM + bacteria) to a 96-well black plate containing a final concentration of 50 μM Leu-AMC. Control reactions including substrate only, bacteria only, DOM only, and FSW were measured simultaneously. Data were collected every 5 min for 30 min using a SpectraMax M3 microplate reader (360-nm excitation and 440-nm emission). Before fitting data with a linear model, raw fluorescence units (RFU) were plotted as a function of time to identify any saturating kinetics within the dataset. A standard curve of AMC fluorescence was used to convert RFU to substrate hydrolysis rates (in moles of substrate hydrolyzed per hour) and further normalized to cell concentration for cellular aminopeptidase measurements.

### Data visualization and statistical analysis

Statistical analyses were performed in the open source platform R, version 4.1.2 “Bird Hippie.” Data visualization and plotting were produced using the R package ggplot2 (*80*). Statistical analyses from all tests are summarized in table S1.
