# Supplementary material for: Taxonomically distinct diatom viruses differentially impact microbial processing of organic matter
Source: Sci Adv. 2025 May 2;11(18):eadq5439. doi: 10.1126/sciadv.adq5439 (PMC12047433; doi:10.1126/sciadv.adq5439)
Supplement: Supplementary file 1 — Figs. S1 to S5 Table S1 [file sciadv.adq5439_sm.pdf]

Science Advances
AAAS

# Supplementary Materials for

## Taxonomically distinct diatom viruses differentially impact microbial processing of organic matter

Chana F. Kranzler *et al.*

Corresponding author: Chana F. Kranzler, chana.kranzler@biu.ac.il; Kimberlee Thamatrakoln, thamat@marine.rutgers.edu

*Sci. Adv.* **11**, eadq5439 (2025)
DOI: 10.1126/sciadv.adq5439

**This PDF file includes:**

Figs. S1 to S5
Table S1

**Fig. S1.**

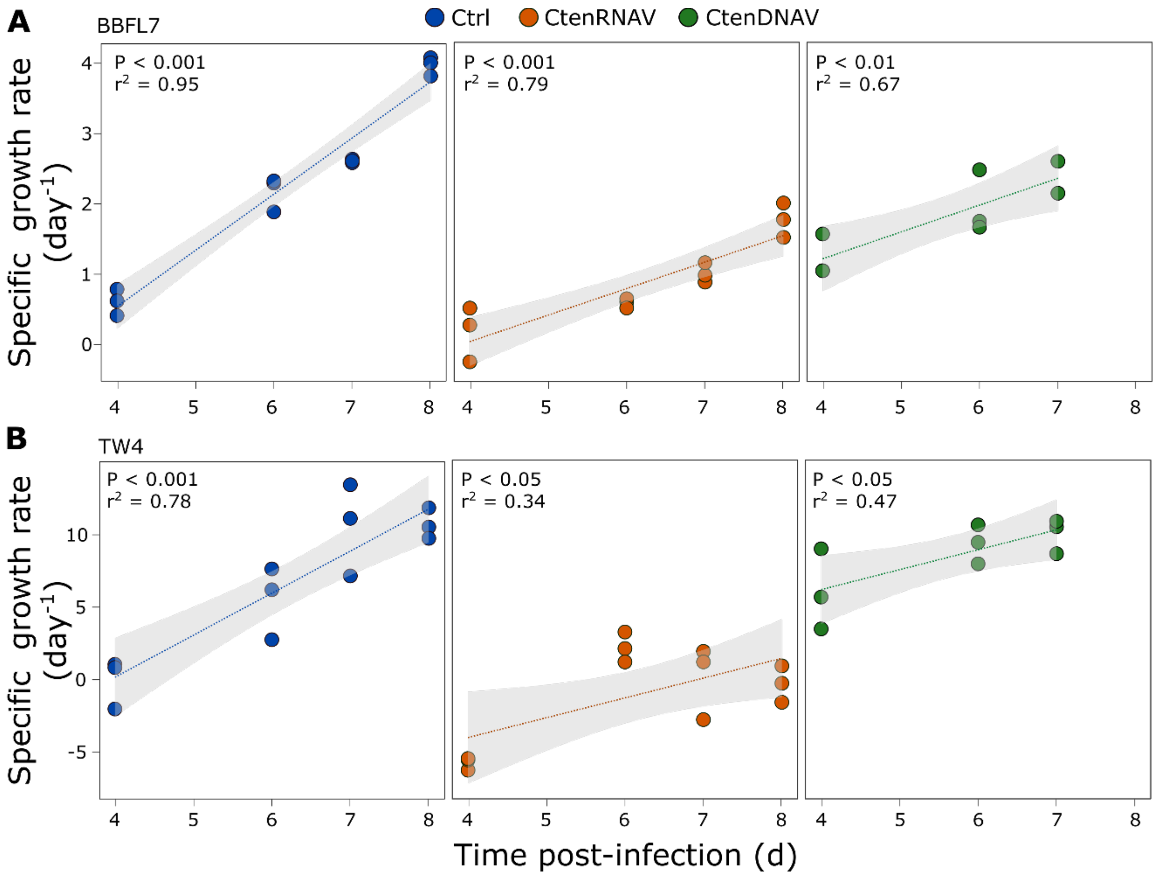


**Figure S1. Correlation between eDOM collection time and bacterial growth.** Relationship between specific growth rates ($\mu$; day$^{-1}$) of **A,** BBFL7 and **B,** Tw4 and collection time of diatom-derived eDOM from *C. tenuissimus* in uninfected control cultures (blue, left panel) and throughout the course of infection with CtenRNAV (orange, middle panel) and CtenDNAV (green, right panel). Lines of best fit with 95% confidence intervals (grey shading) are shown describing linear regression analysis with $r^2$ and $P$-value depicted for each fit. See Table S1 for summary of statistical analyses.

**Fig. S2.**

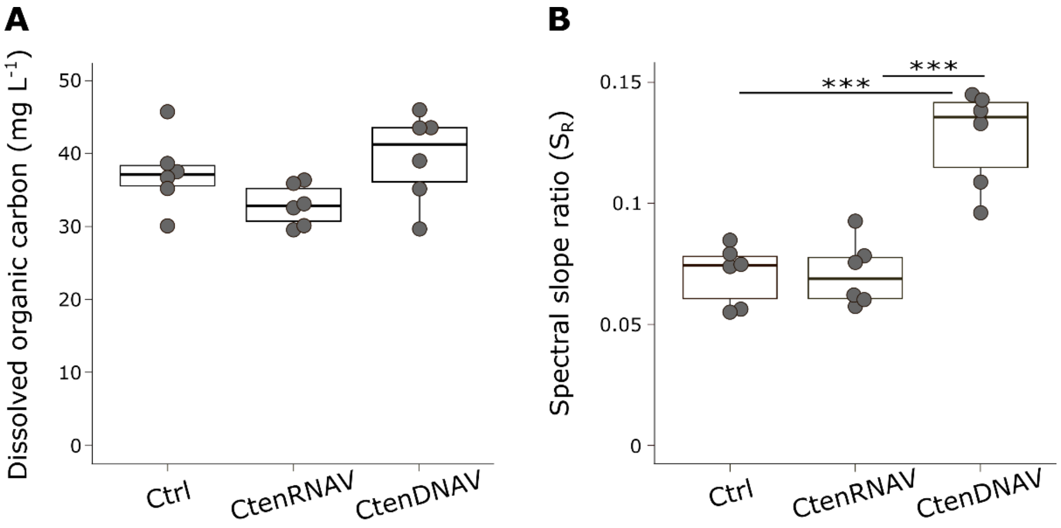


**Figure S2. Characterization of extracellular dissolved organic matter. A,** Dissolved organic carbon concentration (mg $L^{-1}$) and **B,** spectral slope ratios ($S_R$; $S_{275\text{-}295}$:$S_{350\text{-}400}$) in eDOM fractions collected at 4 and 6 dpi. Boxes depict median (line), upper and lower quartiles; whiskers denote values 1.5× the interquartile range. ***$P < 0.001$, ANOVA, Tukey's HSD post-hoc test. See Table S1 for summary of statistical analyses.

**Fig. S3.**

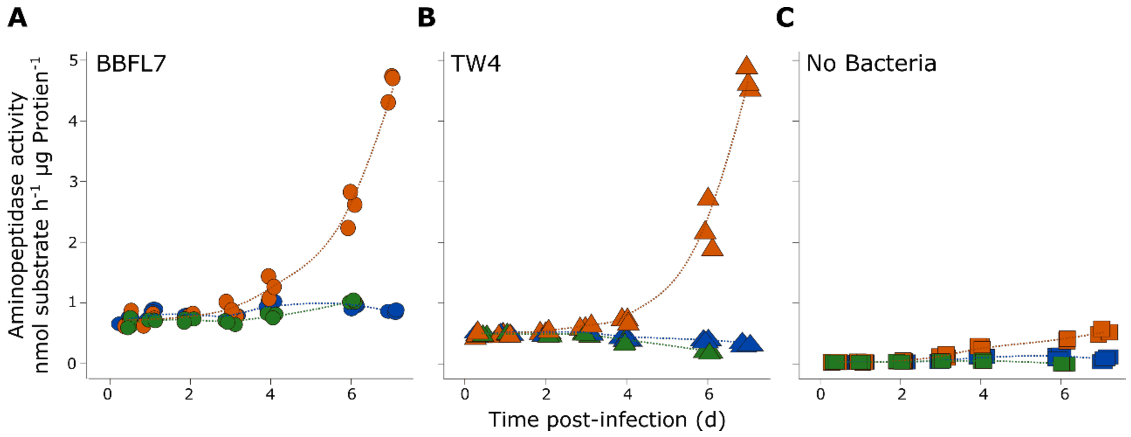


**Figure S3. Quantitative comparison between bacterial and diatom-derived aminopeptidase activity.** Protein-normalized aminopeptidase specific activity (nmol AMC $h^{-1}$ µg $protien^{-1}$) in **A,** BBFL7 and **B,** Tw4 upon exposure to intracellular dissolved organic matter (iDOM) in comparison with **C,** inherent, background aminopeptidase activity in iDOM samples in uninfected *C. tenuissimus* cultures (blue symbols) and cultures infected with CtenRNAV (orange symbols) and CtenDNAV (green symbols) over a time course of viral infection.

**Fig S4.**

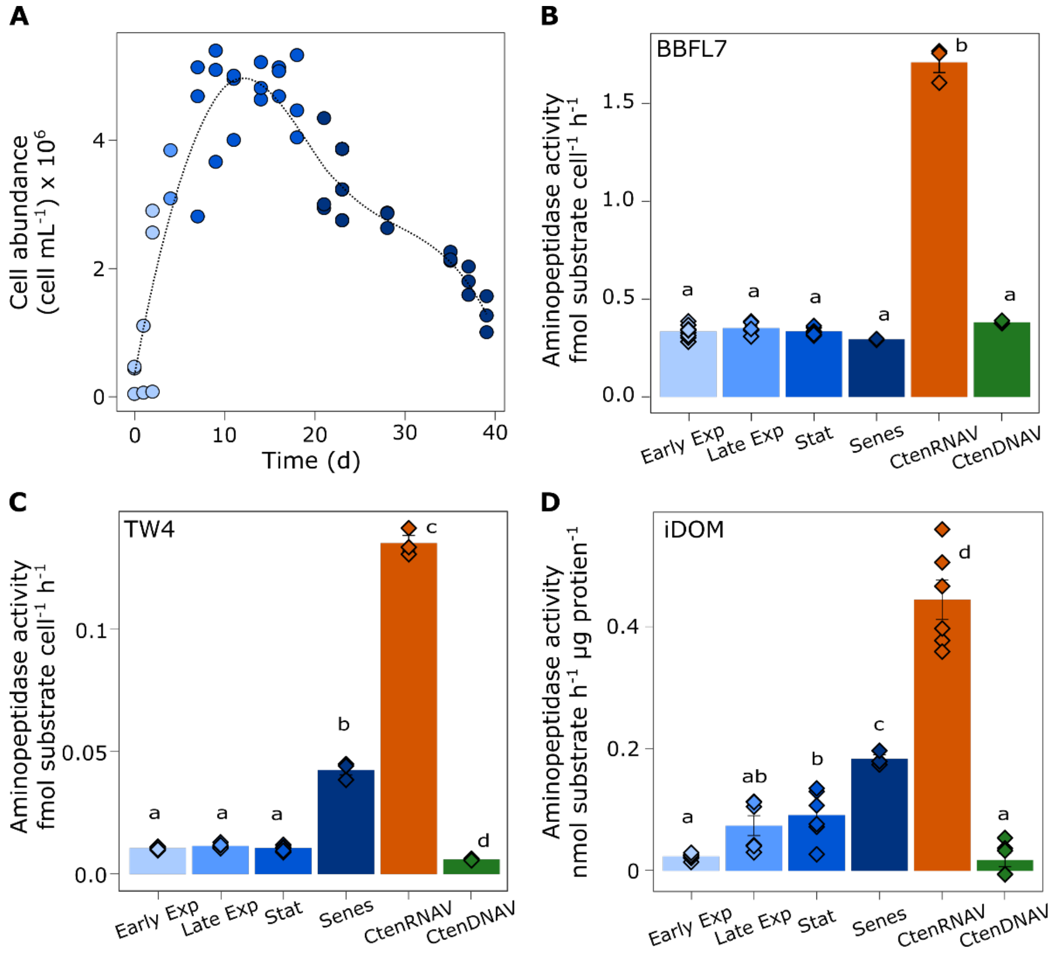


**Figure S4. Assessing impacts of DOM derived from senescent *C. tenuissimus* cells on aminopeptidase activity. A,** Cell concentration was monitored over 39 days to assess growth, aging and senescence in *C. tenuissimus*. Intracellular DOM (iDOM) was collected from visibly senescent cultures after 23 days of growth for downstream experiments to compare with iDOM collected during exponentially growing and stationary phase Ctrl cultures (Fig. 1). Cell-specific aminopeptidase activity in **B,** BBFL7 and **C,** Tw4 (fmol substrate cell$^{-1}$ h$^{-1}$) upon exposure to intracellular dissolved organic matter (iDOM) and in **D,** iDOM extracts (nmol substrate h$^{-1}$ µg protien$^{-1}$) collected during early exponential (day 0 – 2), late exponential (day 3-4) stationary phase (day 6-7) and senescent (day 23) Ctrl cultures (blue) as compared to CtenRNAV (7 dpi, orange) and CtenDNAV (6 dpi, green, Fig. 4) treatments. Mean and standard error of triplicate incubations are shown along with individual replicates (diamonds, n = 3 - 6). Letters denote statistically significant groups ($P < 0.05$), ANOVA and Tukey's HSD post-hoc test. See Table S1 for summary of statistical analyses.

**Fig S5.**

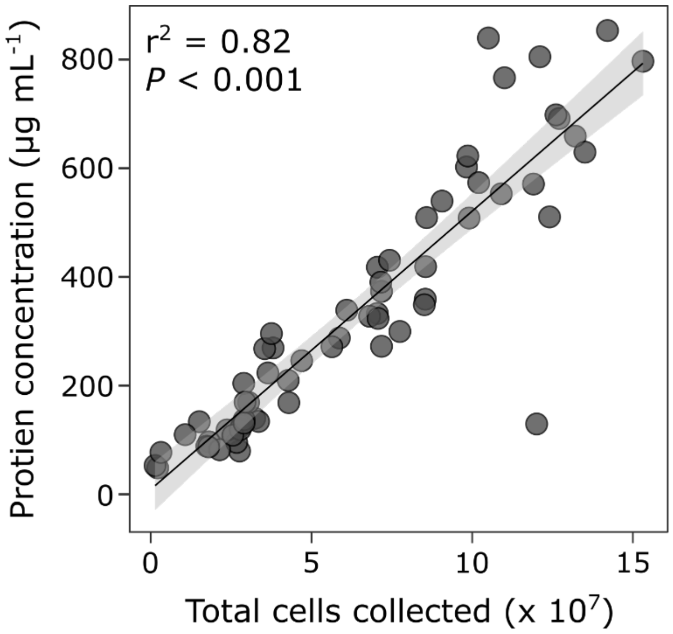


**Supplementary Figure 5. Correlation between protein concentration and cell number.** Relationship between protein concentration (μg mL$^{-1}$) in iDOM extracts as a function of the total number of cells collected in each sample throughout the time course of infection across all treatments. Lines of best fit with 95% confidence intervals (grey shading) are shown describing linear regression analysis ($r^2$ = 0.82, $P$ < 0.001, Table S1).

# Table S1.

**Statistics Summary**

| Figure | Test | Interaction | *F*-statistic | *P*-value | $r^2$ | *r* |
|---|---|---|---|---|---|---|
| 2a | two-way ANOVA | Growth Rate ~Treatment*Time | $F_{(2,26)} = 15.7$ | $P < 0.001$ | | |
| 2b | two-way ANOVA | Growth Rate ~Treatment*Time | $F_{(5,22)} = 4.48$ | $P < 0.01$ | | |
| 2c | ANOVA | Growth Rate ~Treatment | $F_{(4,10)} = 48.1$ | $P < 0.001$ | | |
| 2d | ANOVA | Enzyme Rate ~Treatment | $F_{(2,30)} = 31.2$ | $P < 0.001$ | | |
| 2e | ANOVA | Enzyme Rate ~Treatment | $F_{(2,30)} = 9.97$ | $P < 0.001$ | | |
| 2f | ANOVA | Enzyme Rate ~Treatment | $F_{(2,21)} = 9.90$ | $P < 0.001$ | | |
| 3a | two-way ANOVA | Enzyme Rate ~Treatment*Time | $F_{(11,40)} = 124$ | $P < 0.001$ | | |
| 3b | two-way ANOVA | Enzyme Rate ~Treatment*Time | $F_{(11,40)} = 215$ | $P < 0.001$ | | |
| 4a | two-way ANOVA | Enzyme Rate ~Treatment*Time | $F_{(10,38)} = 13.3$ | $P < 0.001$ | | |
| 4b | ANOVA | Enzyme Rate ~Treatment | $F_{(2,41)} = 32.4$ | $P < 0.001$ | | |
| 4c;Ctrl | Linear Regression | Extracellular Enzyme Rate ~ Intracellular Enzyme Rate | $F_{(1,19)} = 1.75$ | $P = 0.201$; **ns** | 0.036 | 0.29 |
| 4c;CtenRNAV | Linear Regression | Extracellular Enzyme Rate ~ Intracellular Enzyme Rate | $F_{(1,19)} = 7.153$ | $P < 0.05$ | 0.24 | 0.52 |
| 4c;CtenDNAV | Linear Regression | Extracellular Enzyme Rate ~ Intracellular Enzyme Rate | $F_{(1,16)} = 1.671$ | $P = 0.214$; **ns** | 0.04 | -0.31 |
| S1a; Ctrl | Linear Regression | Growth rate ~ Time post infection | $F_{(1,10)} = 232$ | $P < 0.001$ | 0.95 | 0.98 |
| S1a; CtenRNAV | Linear Regression | Growth rate ~ Time post infection | $F_{(1,10)} = 42.5$ | $P < 0.001$ | 0.79 | 0.90 |
| S1a; CtenDNAV | Linear Regression | Growth rate ~ Time post infection | $F_{(1,6)} = 15.3$ | $P < 0.01$ | 0.67 | 0.85 |
| S1b; Ctrl | Linear Regression | Growth rate ~ Time post infection | $F_{(1,10)} = 41$ | $P < 0.001$ | 0.78 | 0.90 |
| S1b; CtenRNAV | Linear Regression | Growth rate ~ Time post infection | $F_{(1,10)} = 6.69$ | $P < 0.05$ | 0.34 | 0.63 |
| S1b; CtenDNAV | Linear Regression | Growth rate ~ Time post infection | $F_{(1,7)} = 8.11$ | $P < 0.05$ | 0.47 | 0.73 |
| S2a | ANOVA | DOC ~ Treatment | $F_{(2,15)} = 2.78$ | $P = 0.09$; ns | | |
| S2b | ANOVA | $S_R$ ~ Treatment | $F_{(2,15)} = 25.9$ | $P < 0.001$ | | |
| S4b | ANOVA | Enzyme Rate ~Treatment | $F_{(3,14)} = 52.0$ | $P < 0.001$ | | |
| S4c | ANOVA | Enzyme Rate ~Treatment | $F_{(3,14)} = 103$ | $P < 0.001$ | | |
| S4d | ANOVA | Enzyme Rate ~Treatment | $F_{(5,30)} = 93.1$ | $P < 0.001$ | | |
| S5 | Linear Regression | Protien concentration ~ Cells collected | $F_{(1,61)} = 290$ | $P < 0.001$ | 0.82 | 0.91 |

**Supplementary Table 1. Summary of statistical analyses reported throughout the manuscript.** Reported parameters include the statistical test performed (Test), interaction tested (Interaction), *F*-statistic, *P*-value, adjusted $r^2$ ($r^2$) and Pearson coefficient (*r*).
